# Use of alternative bioassays to explore the impact of pyrethroid resistance on LLIN efficacy

**DOI:** 10.1186/s13071-020-04055-9

**Published:** 2020-04-07

**Authors:** Marissa K. Grossman, Shüné V. Oliver, Basil D. Brooke, Matthew B. Thomas

**Affiliations:** 1 Department of Entomology, Pennsylvania State University, University Park, PA USA; 2 Centre for Emerging Zoonotic and Parasitic Diseases, National Institute for Communicable Diseases of the National Health Laboratory Service, Johannesburg, South Africa; 3 Wits Research Institute for Malaria, MRC Collaborating Centre for Multi-disciplinary Research on Malaria, School of Pathology, Faculty of Health Sciences, University of the Witwatersrand, Johannesburg, South Africa

**Keywords:** Insecticide resistance, *Anopheles*, Malaria, Pyrethroids

## Abstract

**Background:**

There is substantial concern that the spread of insecticide resistance will render long-lasting insecticide-treated nets (LLINs) ineffective. However, there is limited evidence supporting a clear association between insecticide resistance and malaria incidence or prevalence in the field. We suggest that one reason for this disconnect is that the standard WHO assays used in surveillance to classify mosquito populations as resistant are not designed to determine how resistance might impact LLIN efficacy. The standard assays expose young, unfed female mosquitoes to a diagnostic insecticide dose in a single, forced exposure, whereas in the field, mosquitoes vary in their age, blood-feeding status, and the frequency or intensity of LLIN exposure. These more realistic conditions could ultimately impact the capacity of “resistant” mosquitoes to transmit malaria.

**Methods:**

Here, we test this hypothesis using two different assays that allow female mosquitoes to contact a LLIN as they host-seek and blood-feed. We quantified mortality after both single and multiple exposures, using seven different strains of *Anopheles* ranging in pyrethroid resistance intensity.

**Results:**

We found that strains classified as 1×-resistant to the pyrethroid insecticide deltamethrin in the standard WHO assay exhibited > 90% mortality over 24 h following more realistic LLIN contact. Mosquitoes that were able to blood-feed had increased survival compared to their unfed counterparts, but none of the 1×-resistant strains survived for 12 days post-exposure (the typical period for malaria parasite development within the mosquito). Mosquitoes that were 5×- and 10×-resistant (i.e. moderate or high intensity resistance based on the WHO assays) survived a single LLIN exposure well. However, only about 2–3% of these mosquitoes survived multiple exposures over the course of 12 days and successfully blood-fed during the last exposure.

**Conclusions:**

These results suggest that the standard assays provide limited insight into how resistance might impact LLIN efficacy. In our laboratory setting, there appears little functional consequence of 1×-resistance and even mosquitoes with moderate (5×) or high (10×) intensity resistance can suffer substantial reduction in transmission potential. Monitoring efforts should focus on better characterizing intensity of resistance to inform resistance management strategies and prioritize deployment of next generation vector control products.
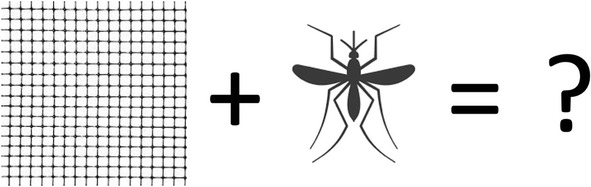

## Background

The prevalence of insecticide resistance in malaria vector populations has been increasing steadily over the past 15 years [1], leading to concerns over widespread failure of insecticide-based vector control measures such as long-lasting insecticidal nets (LLIN) [2, 3]. However, despite these concerns, evidence of LLIN control failure due to resistance is mixed. The most compelling data derive from a randomized controlled trial in an area of high pyrethroid resistance in Tanzania, which showed that LLINs containing a synergist that inhibits the detoxification of pyrethroids by resistant mosquitoes provided improved control of malaria transmission compared with a standard LLIN [4]. Other studies, however, have found that LLINs are still more effective in preventing both clinical and subclinical malaria infections than untreated nets in areas with high pyrethroid resistance [5–8], including a 4-year WHO-coordinated 5-country cohort study that found no association between pyrethroid resistance and malaria incidence or prevalence in the field [9]. While observational studies need to be treated with caution as they cannot control for various factors and tend to only assess personal protection (a modeling analysis suggests that there could be loss of community protection even if levels of personal protection from LLINs remains high [10]), there are clearly complexities in interpreting the epidemiological consequences of resistance [11, 12].

There are a number of reasons why insecticide resistance might not reduce the apparent effectiveness of LLINs. First, LLINs provide a physical barrier that can reduce biting rate regardless of insecticidal activity. Secondly, there may be sub-lethal effects to insecticide exposure, so that when a resistant mosquito contacts insecticide, it might experience a decrease in its ability to blood-feed and host-seek [11], or even incubate the parasite [13, 14]. Thirdly, alleles that confer resistance in mosquitoes might also reduce vector competence in the absence of insecticide exposure [15]. Fourthly, there might be fitness costs to resistance [16–18], which would impact the resistant population’s survival (and therefore they may not survive long enough to potentially transmit the parasite), though this is not always the case [19]. This diversity of effects illustrates the potential for complex interactions between resistance and overall vectorial capacity of mosquito populations [20].

One additional explanation for why resistance has not substantially impacted control is that we might not have reached a tipping point at which resistance is intense enough to hinder control efforts [12]. This possibility raises questions over the way in which resistance is characterized in the field. The standard WHO resistance assay consists of placing up to 25 female mosquitoes in a plastic tube lined with insecticide-treated paper for one hour and then evaluating mortality 24 hours after exposure [21]. The assay uses a diagnostic concentration of insecticide that is twice the lowest concentration determined to cause 100% mortality of a susceptible strain [21]. If the test mosquitoes display less than 90% mortality to this concentration, the population is characterized as resistant. The assay is designed as a surveillance tool to detect the emergence of resistance, and there are numerous efforts underway that collate prevalence data to illustrate the spatial and temporal distribution of resistance [22–24]. However, demonstrating the presence of resistance in field populations is not the same as demonstrating functional significance. In particular, the WHO tube assay exposes young (3–5 day-old), non-blood-fed, and non-infectious mosquitoes to a relatively low diagnostic dose of insecticide. In the field, the mosquitoes responsible for transmission are at least two weeks-old, have had at least one blood meal, and might well have experienced multiple exposures to higher concentrations of insecticides through repeated contact with LLINs. These differences could matter since it has been shown that phenotypic expression of resistance declines with mosquito age [25–27], can be affected by blood-feeding status [28, 29], and can decrease with multiple exposures [30]. Recently, WHO expanded the scope of the standard tube assay to measure the intensity of resistance by increasing the diagnostic dose to 5× and 10× [21]. While these doses add more information on the nature of resistance, the operational relevance of moderate (5×) or high (10×) intensity resistance in a tube test remains unclear.

While the WHO has developed the cone test and the tunnel test to directly assess the bioefficacy of LLINs [31], these tests also fail to better approximate field conditions. The cone test, which exposes groups of five mosquitoes directly to an LLIN, also dictates the use of young, non-blood-fed mosquitoes, and forces exposure through a very confined area instead of allowing the mosquito to naturally contact the net. The tunnel test, on the other hand, allows the mosquito to host-seek and blood-feed, though it uses a rodent host, which is not the preferred host of the anthropophilic *Anopheles* malaria vectors [32]. At present, there are no existing tools that can fully evaluate the response of resistant mosquitoes as they naturally contact a LLIN.

Here, we quantify the impacts of a standard LLIN on strains of *Anopheles* spp. with different resistance intensities under assay conditions that allow for single or multiple contacts with the LLIN as the mosquitoes host-seek and blood-feed. The aim is to better understand the functional significance of 1×, 5× or 10× resistance in terms of likely efficacy failure of an LLIN.

## Methods

### Strain characterization

We used 7 laboratory strains of *Anopheles* spp. ranging from fully susceptible to 10× pyrethroid-resistant according to the WHO tube assays (Table 1). All strains were maintained at the National Institute for Communicable Diseases in Johannesburg, South Africa, with strains maintained as per Hunt et al. [33]. Further strain details are provided in Venter et al. [34].

**Table 1:** Strain characterization

Species	Strain	Origin	Date colonized	Resistance intensity to deltamethrin	Mortality (%)^a^
*An. arabiensis*	KGB	Kanyemba, Zimbabwe	1975	Susceptible reference	–
*An. funestus*	FANG	Calueque, Angola	2002	Susceptible reference	–
*An. arabiensis*	SENN	Sennar, Sudan	1980	Susceptible	> 98
*An. arabiensis*	SENN-DDT	Sennar, Sudan	Selected since 1995	1×	54
*An. gambiae*	TONGS	Tongon, Côte DʼIvoire	2010	1×	91
*An. funestus*	FUMOZ	Maputo, Mozambique	2000	5×	8
*An. funestus*	FUMOZ-R	Maputo, Mozambique	2001	10×	4

Resistance intensity to the pyrethroid deltamethrin was previously measured under standard laboratory conditions with the WHO tube assays [34]

^a^24 h post-exposure to 1× diagnostic dose of deltamethrin

### Experimental design

#### Experiment 1

**Fig. 1:**
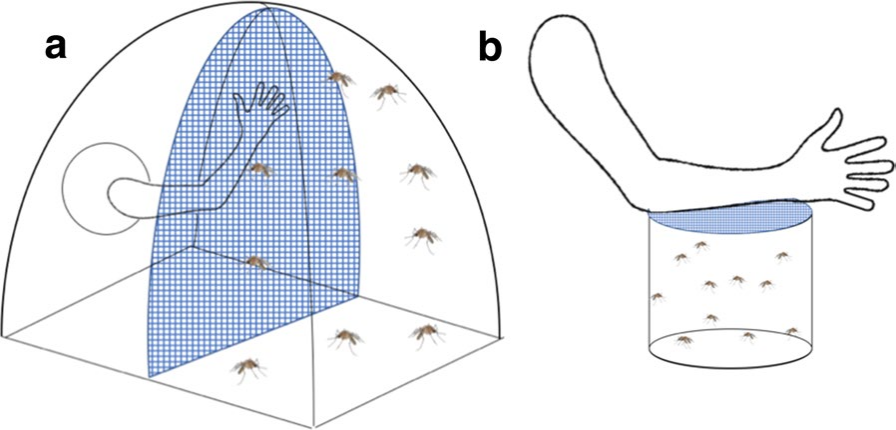
Experimental setup. **a** Experiment 1, the “tent” assay: a Permanet 2.0 divided a BugDorm2120 Insect Rearing Tent into two sections. A human host placed her arm inside of the tent and pressed it against the net, allowing mosquitoes on the other side of the net to obtain a bloodmeal through the net. **b** Experiment 2, the “cup” assay: a 16oz paper cup was covered with a Permanet 2.0. Mosquitoes were released into the cup through a small hole in the net, and the human host placed her arm on top of the net, allowing mosquitoes to obtain a blood meal through the net

As described above, the standard WHO resistance assay involves forced exposure of mosquitoes to a diagnostic dose of insecticide. To simulate more realistic exposure conditions, we suspended either an untreated net or a Permanet 2.0 (a polyester LLIN coated with deltamethrin at 1.8 g/kg) down the middle of a BugDorm2120 Insect Rearing Tent, dividing the tent into two sections (Fig. 1a). Exposures were performed at 25 ± 2 °C and at a relative humidity of 80 ± 5%. A single human host placed her arm inside the tent on one side of the Permanet, pressing her arm against the side of the net, simulating what might happen if a person slept touching an LLIN. We released 20–30 mosquitoes into the tent on the other side of the net from the human host. The net made a functional barrier between the mosquitoes and the host; the only way the mosquito could bite the host was through the net. Mosquitoes that were released into the tent were allowed to host-seek and blood-feed for 20 min. Previous research found that when mosquitoes encounter a LLIN, the majority of activity occurs in the first 10 min, with minimal activity after 30 min [35]. Pilot tests with our experimental set-up revealed that activity greatly reduced after 20 min, so we chose that as the experimental time. During the assay, we counted the number of individual mosquitoes that engaged in host-seeking behavior, defined as flying toward the host and contacting the net, regardless of time spent on the net. It was possible to track individual mosquitoes due to limited flight activity. At the end of 20 min, all mosquitoes were separated by blood-fed status and transferred into holding cups with 10% sucrose solution. Initial mortality, 24-h mortality, and subsequent daily mortality was recorded for 12 days for both blood-fed and non-blood-fed mosquitoes. Surviving mosquitoes at day 12 post-assay were re-released into the tent and allowed to host-seek and blood-feed using the same methods. The timing of this second exposure was designed to coincide with the typical time taken for malaria parasites to complete development within the mosquito (the extrinsic incubation period, EIP) if the mosquito had acquired parasites during the first blood meal [36]. Only those mosquitoes that survive across the EIP and take at least two blood meals can transmit malaria. All assays were conducted in the dark using a red light. The control assays with the untreated net were always conducted first to minimize any risk of insecticide contamination of the human host, who was the same for all experiments and replicates. Four replicates were conducted for each strain and each condition (untreated net vs LLIN). Experiments occurred during October 2017, while tests in Experiment 2 were conducted in February 2018.

#### Experiment 2

The first experiment revealed low recruitment to the host in some mosquito strains. Low recruitment, and therefore low contact with the LLIN, complicated comparison of mortality across strains (note, however, that spatial repellency and/or contact irritancy can be functional properties of an LLIN [37]). In an attempt to address this problem, we conducted a follow-up experiment using much smaller paper cups, measuring 475 ml, as the enclosure (Fig. 1b). Netting was used to cover the opening of the cup and the arm of a volunteer was positioned on top of the netting. Our aim was to allow mosquitoes to contact the netting naturally during host-seeking and blood-feeding as before, but to strengthen the host cues so that the proportion of responders was increased. The same basic procedures were followed as in experiment one, with a few modifications: (i) total experimental time was only 15 min instead of 20 because pilot tests revealed the mosquitoes recruited much faster in the smaller space; (ii) contact with the net, without blood-feeding, could not be seen due to experimental set-up, so only the number of blood-fed mosquitoes was recorded; (iii) the assay was conducted every 3 days for the 12-day experimental period instead of just day one and day 12 to simulate contact with LLINs as mosquitoes attempt to blood-feed across sequential gonotrophic cycles; and (iv) only SENN-DDT, FUMOZ and FUMOZ-R were used for the experiment since the previous assay showed that the other strains suffered close to 100% mortality rapidly after a single exposure. The experiment included four replicate cups per strain, with 20–25 mosquitoes per cup.

### Analysis

A Chi-square test of independence was used to assess the differences in host-seeking and blood-feeding between the LLIN and the untreated net for each mosquito strain. The number of mosquitoes that engaged in host-seeking behavior was defined as the number that contacted the net for any amount of time. To determine the probability of mortality following net exposure, a generalized linear mixed model with a binomial distribution was used with resistance status and treatment (untreated net/LLIN) as predictors. Resistant status was defined as a factor with the categories 0, 1, 5 and 10 to indicate resistance intensity with 0 representing the susceptible strains (KGB, FANG and SENN). Strain was used as a random effect to account for multiple replicates.

Kaplan-Meir survival curves, stratified by treatment, were created to visualize differences in survival following net exposure and a log-rank test was used to determine significant differences in survival between the LLIN and the untreated net. Additionally, Cox proportional hazard models were used to assess the mortality rates given treatment, blood-feeding, and resistance status.

## Results

### Experiment 1: Tent assay

#### Host-seeking and blood-feeding

**Fig. 2:**
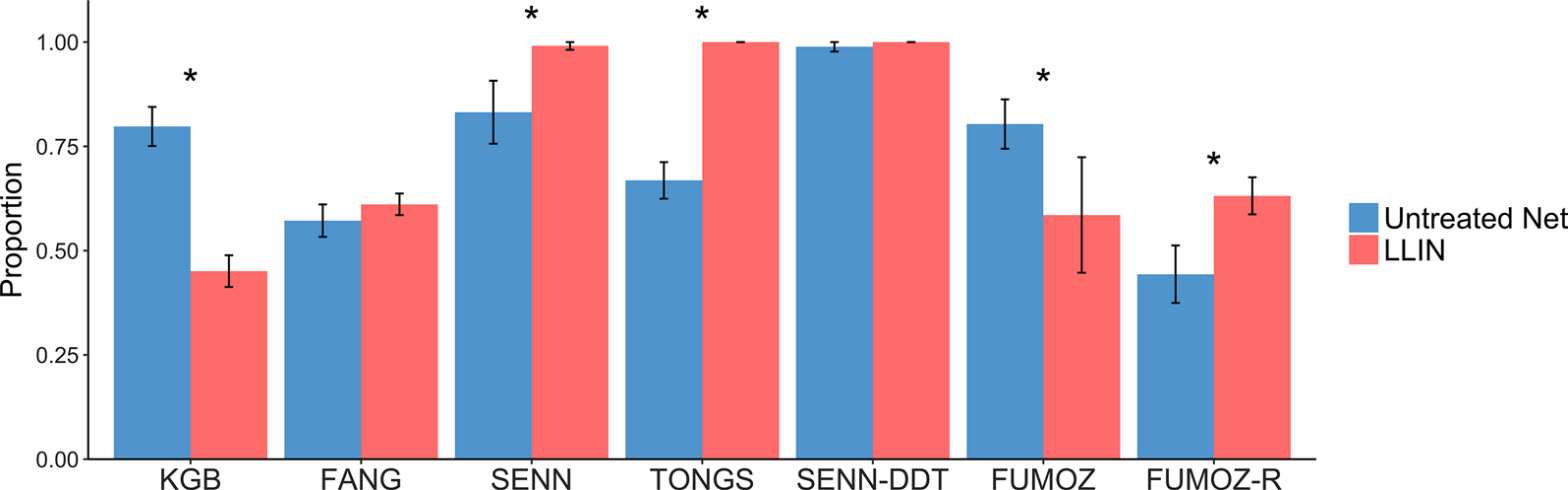
Host-seeking behavior of mosquitoes in the “tent” assay. The bars show the mean (± standard error, SE) proportion of mosquitoes contacting either an untreated net or an LLIN when attempting to feed on a host arm placed adjacent to the net. Mosquito strains on the x-axis are arranged by increasing resistance status. KGB, FANG and SENN are susceptible; TONGS and SENN-DDT are considered 1× resistant; FUMOZ is 5×; FUMOZ-R is 10×. Statistical significance at the alpha level of 0.05 is marked with an *

The proportion of mosquitoes that engaged in host-seeking behavior significantly decreased on the LLIN compared to the untreated net for KGB (Fig. 2; *χ*^2^ = 27.5, *df* = 1, *P* ≤ 0.0001) and FUMOZ (*χ*^2^ = 11.5, *df* = 1, *P* = 0.0007). However, there was significantly more host-seeking on the LLIN for SENN (*χ*^2^ = 16.3, *df* = 1, *P* < 0.0001), TONGS (*χ*^2^ = 28.4, *df* = 1, *P* < 0.0001) and FUMOZ-R (*χ*^2^ = 7.1, *df* = 1, *P* = 0.008), while there was no difference in host-seeking between the LLIN and untreated net for SENN-DDT (*χ*^2^ = 0.01, *df* = 1, *P* = 0.92). FUMOZ-R and FUMOZ displayed the same amount of host-seeking behavior on the LLIN (*χ*^2^ = 0.362, *df* = 1, *P* = 0.547), yet the proportion of FUMOZ-R contacting the untreated net was significantly less than that of FUMOZ (*χ*^2^ = 38.7, *df* = 1, *P* < 0.0001). The other paired lines, SENN and SENN-DDT also showed differences in host-seeking behavior on the untreated net only, with a higher proportion of SENN-DDT contacting the untreated net than SENN (*χ*^2^ = 12.4, *df* = 1, *P* = 0.0004).

**Fig. 3:**
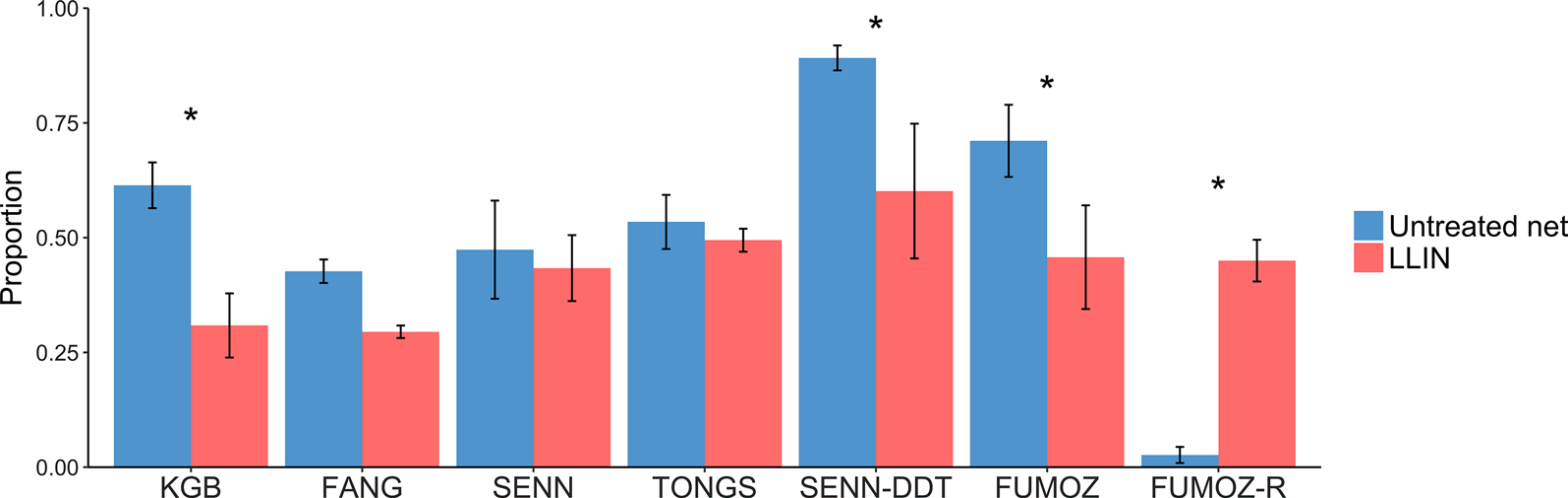
Blood-feeding behavior of mosquitoes in the “tent” assay. The bars show the mean (± standard error, SE) proportion of mosquitoes that contacted either a LLIN or an untreated net and successfully took a blood meal from a human host arm. Mosquito strains on the x-axis are arranged by increasing resistance status. KGB, FANG, and SENN are susceptible; TONGS and SENN-DDT are considered 1× resistant; FUMOZ is 5×; FUMOZ-R is 10×. Statistical significance at the alpha level of 0.05 is marked with an *

All mosquito lines, except for FUMOZ-R, blood-fed less when exposed to the LLIN compared to the untreated net, although this decrease was only significant for KGB (Fig. 3; *χ*^2^ = 21.1, *df* = 1, *P* < 0.0001), SENN-DDT (*χ*^2^ = 14.5, *df* = 1, *P* = 0.0001) and FUMOZ (*χ*^2^ = 12.4, *df* = 1, *P* = 0.0004). FUMOZ-R, however, blood-fed significantly more through a LLIN than through an untreated net (*χ*^2^ = 52.2, *df* = 1, *P* < 0.0001).

#### Short-term mortality and extended survival following contact with a net

**Fig. 4:**
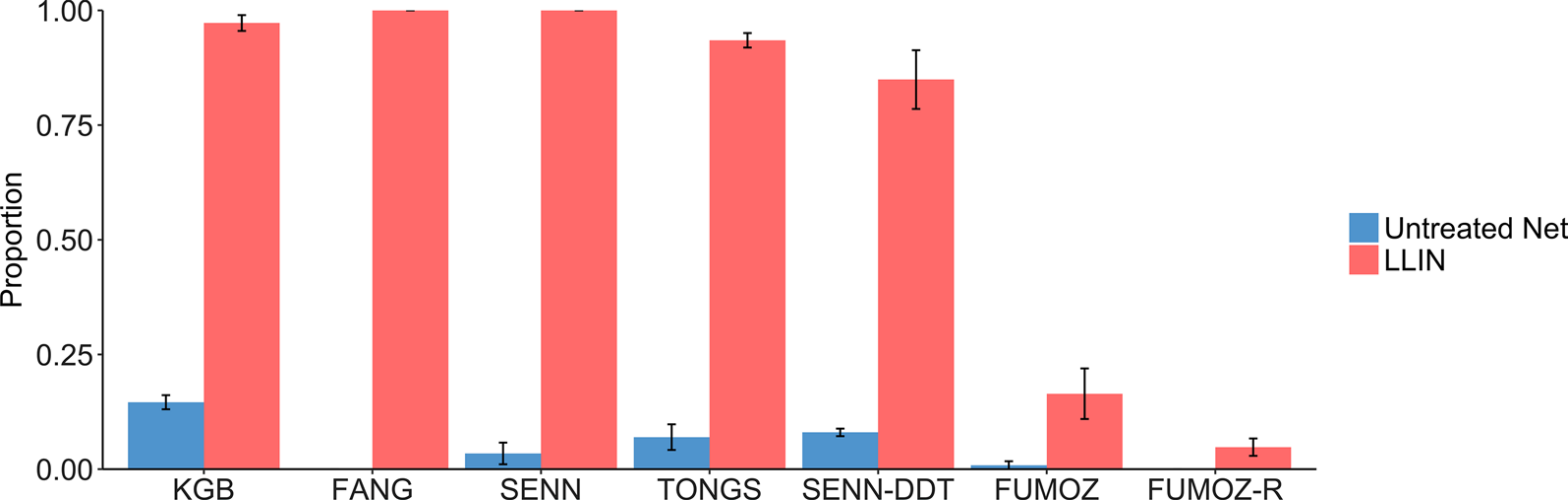
Mortality 24 hours post-exposure. The bars show the mean (± standard error, SE) proportion of mosquitoes that died following exposure to either an LLIN or an untreated net. Mosquito strains on the x-axis are arranged by increasing resistance status. KGB, FANG, and SENN are susceptible; TONGS and SENN-DDT are considered 1× resistant; FUMOZ is 5×; FUMOZ-R is 10×

**Fig. 5:**
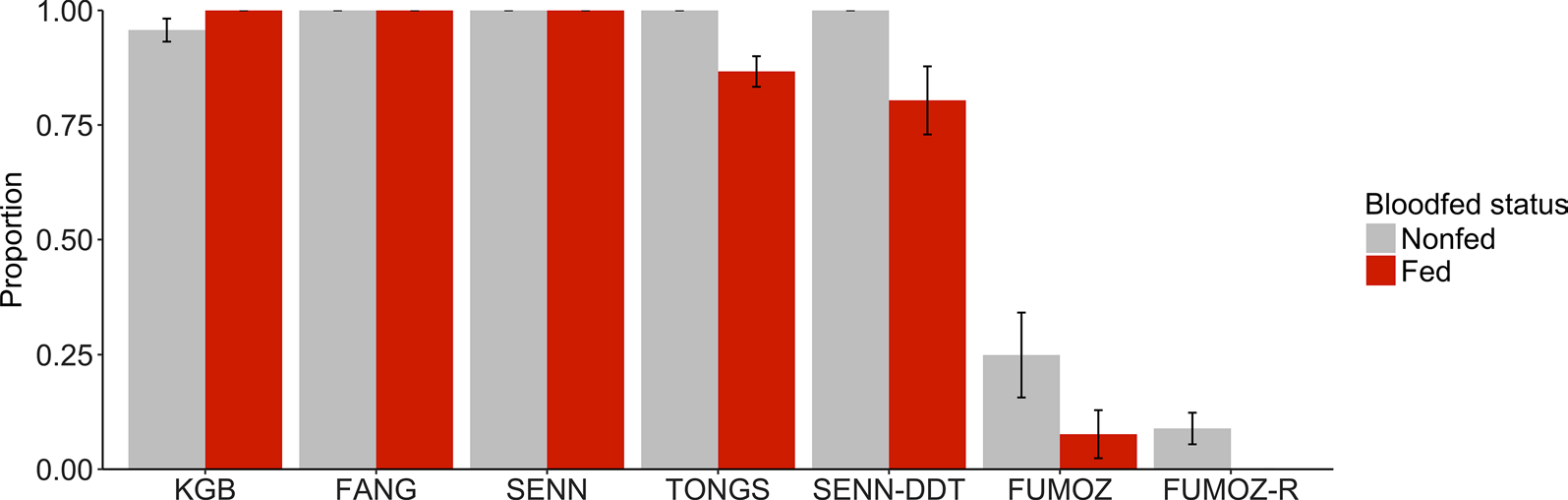
Mortality 24 hours post-exposure on the LLIN based on blood-fed status. The bars show the mean (± standard error, SE) proportion of mosquitoes that died following either a successful or unsuccessful attempt to blood-feed on a human host arm while being exposed to an LLIN. Mosquito strains on the x-axis are arranged by increasing resistance status. KGB, FANG, and SENN are susceptible; TONGS and SENN-DDT are considered 1× resistant; FUMOZ is 5×; FUMOZ-R is 10×

For all mosquito lines there was negligible 24-h mortality following exposure to an untreated net (range 0–12%). The susceptible strains, KGB, SENN and FANG, displayed 97 ± 1.7% (KGB) and 100% (SENN and FANG) mortality at 24 h post-contact with the LLIN. TONGS and SENN-DDT, 1× resistant strains according to the WHO tube assay, had 93.5 ± 1.6% mortality and 84.9 ± 6.4% mortality, respectively (Fig. 4). However, the level of mortality was dependent on whether mosquitoes had blood-fed during the LLIN exposure or not. Mortality of blood-fed TONGS and SENN-DDT was 86.7 ± 3.3% and 80.3 ± 7.4%, respectively, compared to 100% mortality for those that did not blood-feed (Fig. 5). The more resistant FUMOZ and FUMOZ-R exhibited substantially lower overall mortality (16.4 ± 5.5% and 4.8 ± 1.9%, respectively). Again, mosquitoes that had blood-fed suffered lower mortality than those that had not taken a blood meal (7.6 ± 5.2% compared with 24.9 ± 9.3% for FUMOZ and 0% compared with 8.9 ± 3.4% for FUMOZ-R).

The odds of mosquito mortality after one exposure to the LLIN were 2.8 (95% CI: 1.2–6.5) lower for 1× resistant mosquitoes compared to susceptible, 149 (95% CI: 53.6–459) times lower for 5× resistant mosquitoes, and 592 times (95% CI: 189–2194) lower for 10× mosquitoes compared to susceptible, indicating that resistance, as classified by the WHO criteria, does increase survival dramatically (Table 2). Additionally, the odds of mortality of any mosquito following contact with an LLIN, even after accounting for resistance, were 332 (95% CI: 186–635) times higher than following contact with an untreated net, indicating that the insecticide still has an appreciable effect even in the presence of resistance (Table 2).

**Table 2:** General linear mixed effect model assessing the impact of resistance intensity and the experimental treatment on mosquito mortality

Fixed effects	Estimate	SE	*Z*-value	*P-*value
Intercept	− 2.39	0.24	− 9.84	< 0.0001
LLIN	5.81	0.31	18.65	< 0.0001
1× Resistance	− 1.02	0.39	− 2.62	0.0087
5× Resistance	− 5.00	0.48	− 10.41	< 0.0001
10× Resistance	− 6.38	0.58	− 10.99	< 0.0001

*Notes*: Resistance was defined as a factor, with the levels starting at susceptible (the reference) to 1×, 5× and 10×. Experimental treatment was either the LLIN or the untreated net, which was the reference. Mosquito strain was used a random effect to account for multiple replicates per strain (*n* = 54, Groups = 7, Variance = 0.064, Std. Dev = 0.253)

*Abbreviation*: SE, standard error

**Fig. 6:**
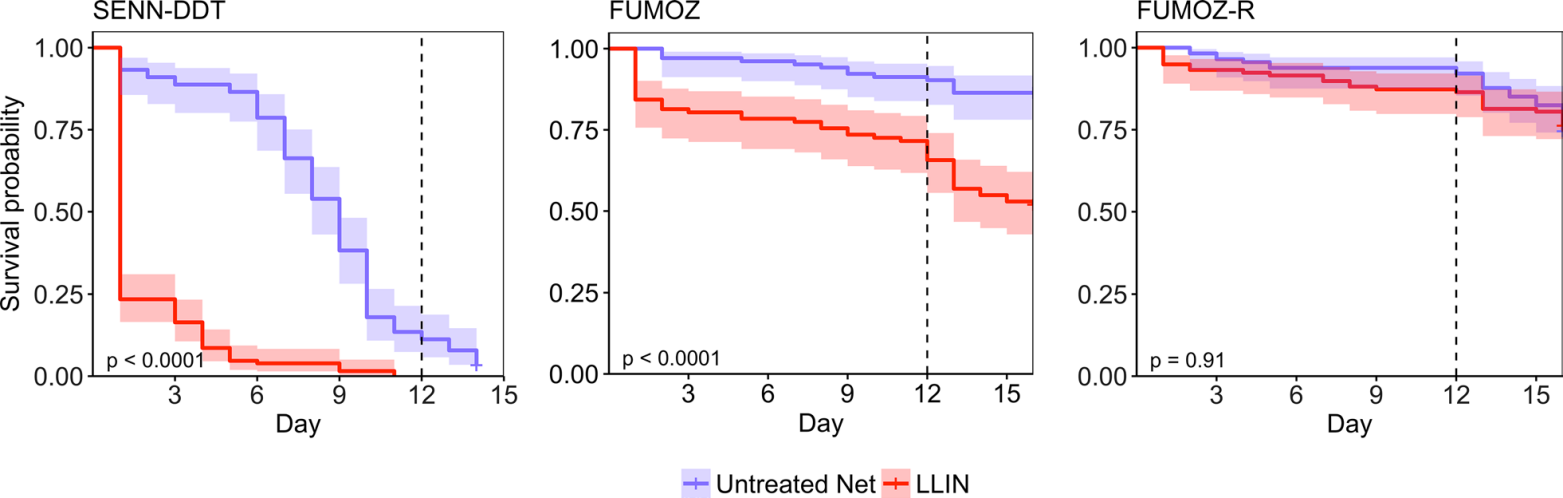
Survival post-exposure. Exposures occurred on day 0 and day 12, although only FUMOZ and FUMOZ-R were exposed on day 12 due to poor survival of SENN-DDT. Shaded areas represent 95% confidence intervals. The *P*-value listed is based on the log-rank test to determine differences in the survival curves for the untreated net and LLIN

Over the course of the entire 12-day experimental period, the overall mortality (regardless of intensity of resistance) for SENN-DDT, FUMOZ and FUMOZ-R was 2.0 (95% CI: 1.6–2.5) times higher if exposed to the LLIN than the untreated net (Fig. 6; Cox PH, *z* = 6.27, *P* < 0.0001). On the LLIN, SENN-DDT suffered a mortality rate that was 11.7 times (95% CI: 7.7–17.7) higher than FUMOZ (Cox PH, *z* = − 11.61, *P* < 0.0001) and 29.1 (95% CI: 17.8–47.7) times higher than FUMOZ-R (Cox PH, *z* = − 13.45, *P* < 0.0001).

Of the 6 strains tested, only FUMOZ and FUMOZ-R survived all 12 days post-exposure (to reach 17 days-old) when exposed to the LLIN (Fig. 6). Of the 64.3 ± 6.1% of FUMOZ and 85.1 ± 7.1% of FUMOZ-R that survived 12-days post LLIN exposure, only 58.8 ± 9.8% of FUMOZ and 40.3 ± 7.6% of FUMOZ-R contacted the net with only 30.4 ± 6.5% of FUMOZ and 7.8 ± 3.3% of FUMOZ-R blood-feeding during the second exposure assay. These low rates may be due to age rather than previous exposure: when 17-day-old FUMOZ-R mosquitoes that had no previous exposure were used for the assay on the LLIN, only 20.6 ± 5.3% contacted the net and 3.7 ± 0.07% blood-fed.

### Experiment 2: Cup assay

#### Blood-feeding

**Fig. 7:**
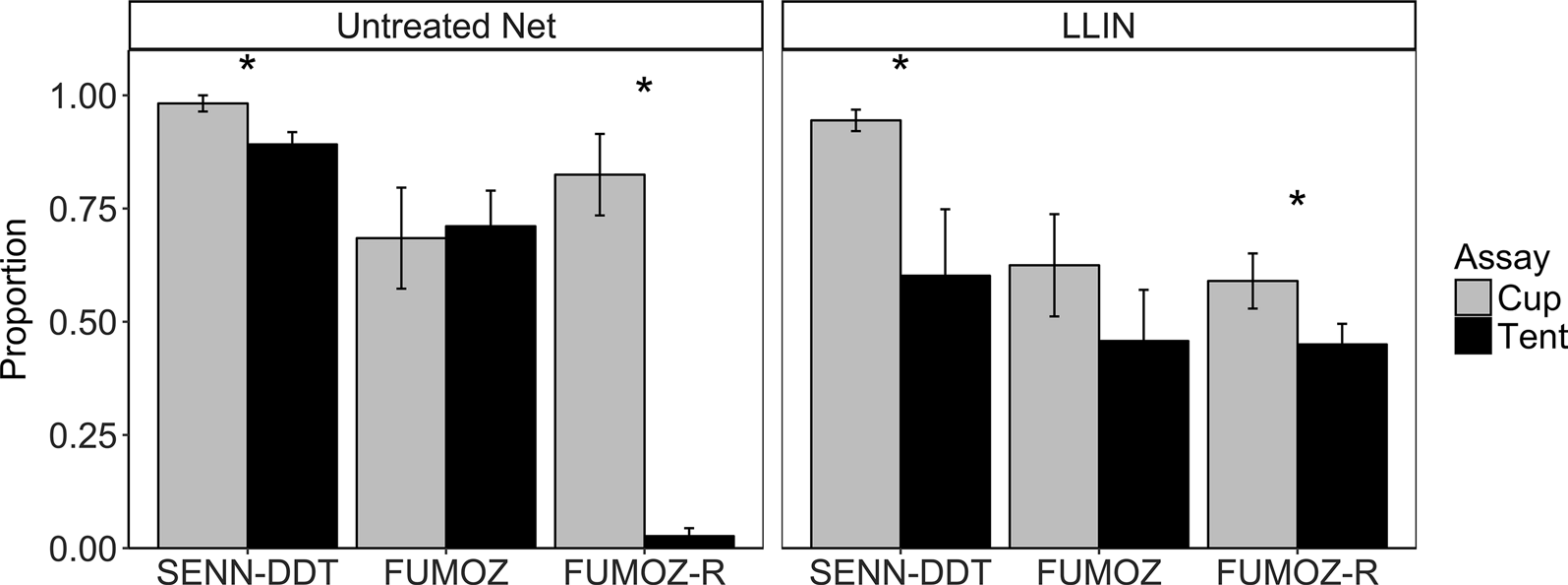
Blood-feeding behavior of mosquitoes in both the tent assay and the cup assay. The bars show the mean (± standard error, SE) proportion of mosquitoes that contacted either a LLIN or an untreated net and successfully took a blood meal from a human host arm. Mosquito strains on the x-axis are arranged by increasing resistance status. KGB, FANG, and SENN are susceptible; TONGS and SENN-DDT are considered 1× resistant; FUMOZ is 5×; FUMOZ-R is 10×. Statistical significance at the alpha level of 0.05 is marked with an *

The cup assay increased the proportion of mosquitoes that blood-fed compared to the tent assay on both the LLIN and untreated net for SENN-DDT and FUMOZ-R (Fig. 7). There were no differences between the proportion of mosquitoes that blood-fed on the LLIN compared to the untreated net for the cup assay except for FUMOZ-R, which blood-fed significantly less on the LLIN than on the untreated net (*χ*^2^ = 10.94, *df* = 1, *P* = 0.0009). Contact rates with the nets could not be observed due to the nature of the cup assay (the arm placed on top of the paper cup obscured behavioral observations).

#### Short-term mortality and extended survival following contact with a net

The proportion of mosquitoes that died 24 h after exposure to the LLIN in the cup assay was 81.1% (± 7.5%) for SENN-DDT, 55.7% (± 12.7%) for FUMOZ, and 27.1% (± 11.4%) for FUMOZ-R. All strains exhibited little to no mortality 24 hours following exposure to an untreated net.

**Fig. 8:**
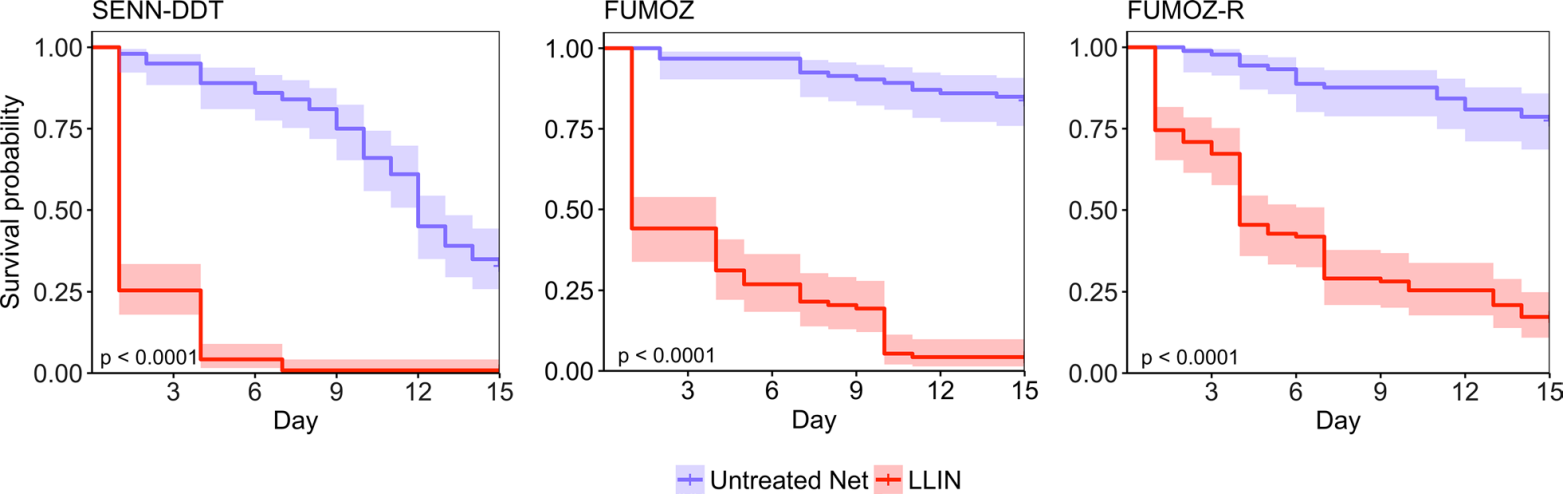
Survival curves given multiple exposures. Exposures occurred on days 0, 3, 6, 9 and 12, and survival was tracked daily. Shaded areas represent 95% confidence intervals. The *P*-value listed is based on the log-rank test to determine differences in the survival curves for the untreated net and LLIN

SENN-DDT, FUMOZ, and FUMOZ-R experienced 5 total exposures on either the untreated net or LLIN over the course of 12 days. All strains suffered significantly higher mortality on the LLIN compared to the untreated net (Fig. 8; SENN-DDT: Log-rank test, *χ*^2^ = 180, *P* < 0.0001, FUMOZ: Log-rank test, *χ*^2^ = 156, *P* < 0.0001; FUMOZ-R: Log-rank test, *χ*^2^ = 82.1, *P* < 0.0001). Controlling for the effect of the LLIN and the mosquito strain, mosquitoes that blood-fed at least once during the 5 exposures had a significantly reduced mortality rate compared to those that did not feed at all (Cox PH, Hazard Ratio = 0.28, 95% CI: 0.21–0.36, *P* < 0.0001). When accounting for blood-feeding, the mortality rate of all strains was 10.5 times higher on the LLIN than the untreated net (Cox PH, Hazard Ratio = 10.52, 95% CI: 8.12–13.58, *P* < 0.0001). Additionally, the mortality rate of FUMOZ and FUMOZ-R was 3.3 and 5.6 times, respectively, lower than SENN-DDT, accounting for LLIN exposure and blood-feeding (Cox PH; FUMOZ; Hazard Ratio = 0.31, 95% CI: 0.24–0.40, *P* < 0.0001; FUMOZ-R: Hazard Ratio = 0.18, 95% CI: 0.14–0.23, *P* < 0.0001).

The proportion of each population that was able to survive the 12-day interval between the first and last exposure on the LLIN and blood-feed during both exposures was minimal, ranging between 0.9–3.2% (Table 3). This was significantly smaller than the proportion that survived and blood-fed during the initial and final exposures on the untreated net (Fisher’s Exact test, *P* < 0.0001 for all strains).

**Table 3:** Proportion of the population that survived and blood-fed on both initial and final exposure

Colony	Initial *n*		*n* on day 12		Individuals that fed on days 0 and 12 (% of initial population)	
	LLIN	Untreated net	LLIN	Untreated net	LLIN	Untreated net
SENN-DDT	104	105	1	46	1 (0.9)	23 (21.9)
FUMOZ	95	101	4	81	3 (3.2)	22 (21.7)
FUMOZ-R	106	94	20	77	2 (1.8)	20 (21.3)

## Discussion

Current understanding of the distribution of resistance derives from extensive use of the standardized WHO discriminating dose assay. It is an intuitive assumption that mosquito populations defined as “resistant” to an insecticide will impact the utility of that insecticide in vector control. We used two novel assays to better understand the possible functional significance of resistance: a “tent” assay, which gave mosquitoes ample space to host-seek (and better approximated natural conditions), and a “cup” assay, which placed the mosquitoes in closer proximity to the host. Following a single exposure to a LLIN in the tent assay, two strains that were categorized as 1× resistant according to the standard WHO tube assays, one *A. gambiae* (TONGS) and the other *A. arabiensis* (SENN-DDT), exhibited greater than 90% mortality 24-hours post-exposure. While there are many differences between our assay and the standard WHO tube test that could produce these results, perhaps the most important factor may be the dose and exposure time. The WHO tube test exposes mosquitoes to insecticide-impregnated papers that are a “diagnostic” dose, i.e. twice the dose required to kill 100% of a susceptible laboratory population, which is 0.05% deltamethrin for *Anopheles*, for an hour. Our assay, on the other hand, used a Permanet 2.0 LLIN, with a much higher dose of 1.8 g/kg (0.18%), and for up to 20 minutes, with the contact time decided by the individual mosquito. The exact bioavailability of the insecticide on the LLIN surface is unclear, but given the potential increase in effective dose, it is perhaps not a surprise that the mosquitoes characterized as 1× resistant died in our assay. While we cannot directly compare our assay with the WHO tube test, we want to highlight that the WHO test tells us very little about what a mosquito may be experiencing in the field, so resistance results abstracted from the assay might not actually correlate to its mortality when it encounters a bednet. This is not a criticism of the WHO tests as they are not designed to estimate the functional impact of resistance. Nonetheless, from an operational perspective, there is a need to understand the epidemiological significance of resistance.

Interestingly, the 1× population SENN-DDT had only 80% mortality in the cup assay, consistent with the WHO assay results. These findings are likely due to blood-feeding; individuals that blood-fed during exposure in both assays had significantly lower mortality than those that did not, and more individuals blood-fed in the cup assay than in the tent assay. In fact, 100% of non-blood-fed individuals died following exposure in both assays. The 5× and 10× resistant mosquito strains used in both assays showed this effect too (susceptible strains died regardless), suggesting that blood-feeding potentially rescues resistant individuals from mortality following insecticide exposure. Other studies have also shown that blood-fed females had greater survival than non-fed females in the standard WHO assays [29, 38, 39], with multiple blood-feeding enhancing the effect [28]. If blood-feeding does indeed have a “rescue effect” on mortality when exposed to insecticides, as our data suggest, then this could be problematic for malaria transmission given that only blood-fed females can acquire the parasite.

However, we also found evidence of blood-feeding inhibition due to the LLIN, which could temper the effect of decreased mortality with a blood meal. In four of the seven strains tested (SENN, SENN-DDT, TONGS and FANG), a lower proportion of mosquitoes that contacted the net blood-fed when exposed to an LLIN than to an untreated net. Studies have shown that deltamethrin-treated nets reduced blood-feeding of *Anopheles*, suggesting that this inhibition could be due to contact irritancy [37, 40]. In a similar study to ours, Glunt et al. [11] also found blood-feeding inhibition in SENN-DDT (1×), FUMOZ (5×), and FUMOZ-R (10×) when mosquitoes were given access to a host after LLIN exposure (and not during exposure). Surprisingly, our results show a much higher feeding compliance in FUMOZ-R on the LLIN than on the untreated net in the tent assay, for which we do not have a good explanation. Studies have found that *Anopheles* with the knock down resistance (*kdr*) allele are more attracted to a LLIN than an untreated net [41, 42], but FUMOZ-R and FUMOZ do not have *kdr* as their resistance is driven by metabolic mechanisms only (see Venter et al. [34] for details). Perhaps these mechanisms also cause an attraction to pyrethroids, though we are not aware of research on this subject.

In both assays, the 5× (FUMOZ) and 10× (FUMOZ-R) resistant strains survived a single exposure extremely well. Their survival is not just because only 50–60% contacted the LLIN; the individuals that blood-fed, which necessarily contacted the net, survived better than their non-fed counterparts. Additionally, the susceptible strains, KGB and FANG, had similar relatively low contact rates with the LLIN, yet 97–100% of them died. The mortality of those mosquitoes that did not contact the net for the susceptible strains could be due to either a spatial effect of the LLIN (for which we can find little information in the literature), or because when we were removing mosquitoes from the assay, it prompted mosquito flight and those that did not previously encounter the net ended up contacting it during this time.

Even though the 5× and 10× populations survived a single LLIN exposure well, and despite the potential “rescue effect” of blood-feeding, our results suggest that it is possible that multiple exposures drive down survival sufficiently to substantially impede transmission potential. Malaria transmission depends on the survival of the mosquito from the time it becomes infected during a blood meal to beyond the extrinsic incubation period (EIP) of the parasite. Given a single exposure, none of the 1×-resistant females survived the typical 10–14 day EIP, meaning that they would be unable to transmit parasites. The 5×-resistant females exposed to the LLIN survived the EIP, but significantly less so than on the untreated net, suggesting that the insecticide-treated net is still having some effect beyond a simple barrier. In contrast, the vast majority of the 10×-resistant females survived the EIP with no difference between the LLIN and untreated net, indicating that the single insecticide exposure had no effect. However, with multiple exposures simulating repeat contact with an LLIN over sequential feeding cycles (mosquitoes are anticipated to blood-feed every 2–4 days as this is the duration of the gonotrophic cycle), all strains suffered significant mortality regardless of resistance, and those mosquitoes that survived the EIP exhibited a reduction in blood-feeding, translating to greatly reduced malaria transmission potential.

In summary, our results suggest that realistic contact with an LLIN imposes substantial mortality against 1×-resistant mosquitoes and hence, this level of resistance might have negligible impact on LLIN efficacy in the field. Moreover, with multiple exposures, even 10×-resistant populations suffer greater mortality and reduced blood-feeding on an LLIN compared to an untreated net, suggesting that LLINs are still likely to contribute to control in areas of more intense resistance. We acknowledge that we have used only a limited number of mosquito strains and one type of LLIN, and populations in the field could exhibit a greater range of resistance mechanisms and intensities to different insecticides [43]. Additionally, we used an unused, unwashed LLIN to be consistent with other resistance assays, which also guaranteed maximum efficacy. Data on durability of nets is somewhat mixed: some studies show PermaNet 2.0 to lose efficacy after 5 washes [44], after 15 washes [45], or potentially to show no significant change in efficacy with washing and/or use [46, 47]. However, it might be expected that decay in the active ingredient and/or loss of net integrity will exacerbate the functional effects of resistance. As such, extending the type of research presented here to nets of different age and use history would be an important next step to examine potential interactions between resistance intensity and net durability. Our simplified assay systems also potentially masked more complex mosquito behaviors that could play a role in nature [35]. Nonetheless, in line with the recommendation of WHO [21] and others [2, 3], our study highlights the need for more data on the intensity of resistance, not only to fully understand the functional significance of resistance but also to manage or mitigate the problem.

Next generation LLINs are close to entering operational use. These nets are designed to overcome pyrethroid resistance, either through use of the synergist piperonyl butoxide (PBO), which reduces the capacity of mosquitoes to detoxify the insecticide, or through the addition of alternative insecticides with modes of action distinct from pyrethroids. As a side experiment, we tested one of these next generation LLINs (Permanet 3.0, which combines deltamethrin with PBO) against our resistant strains using the cup assay (see Additional file 1: Text S1 for brief description and summary of results). All strains, including the 10× strain, suffered 100% mortality within 24 hours on the portion of the net containing PBO (Additional file 2: Figure S1). Mosquitoes also bloodfed less on the PBO portion of the Permanet 3.0 compared to the side of the Permanet 3.0 without PBO (Additional file 3: Figure S2) and suffered 100% mortality regardless of bloodfed status (Additional file 4: Figure S3). Other studies have shown broad-scale efficacy of PBO nets [48], with only limited evidence of PBO net failure [49]. At present, these nets are more costly than traditional pyrethroid-only nets, so it would be beneficial to be able to target their distribution into areas where insecticide resistance is most critical. Targeting areas on the basis of 1× resistance has little strategic value as 1× tells us little about the functional significance of resistance. Based on our results, the distribution of 10× resistance could be more informative to prioritize areas, but unfortunately the data on resistance intensity are very limited [22]. For example, the World Health Organization’s Malaria Threats Map of Vector Insecticide Resistance [22] includes 788 entries for tests conducted using the diagnostic dose of pyrethroid insecticide on *Anopheles* mosquitoes for Kenya alone, yet only 10 tests conducted using the intensity bioassay. In other countries, like Sudan, there are simply no data on resistance intensity despite an abundance of data on the diagnostic concentration (756 studies) [22]. Collecting resistance data is a challenge for many national malaria control programmes, but where this is possible, data on resistance intensity could prove valuable to aid in the strategic distribution of next-generation LLINs, which will almost certainly be a key tool for malaria control in the face of increasing resistance.

## Conclusions

Why has resistance not caused a clear failure of malaria control? The results of this study suggest that the current assays may not be characterizing resistance adequately. We found that mortality following LLIN exposure depends on the type of exposure, the blood-feeding status of the female, and the frequency of exposure, all of which are not accounted for in the current WHO tube assay. Furthermore, we found a distinct difference in survival between 1×-, 5×- and 10×-resistant populations, suggesting that intensity of resistance matters. Regardless of resistance intensity, however, we found that the LLIN was still more effective than an untreated net and given multiple exposures, even a 10×-resistant population suffered significant mortality. While the current WHO assays are a convenient starting point for assessing resistance in the field, new assays need to be developed to better characterize resistance in terms of transmission potential. Gaining a better understanding of what resistance means functionally for transmission will ultimately lead to more efficient vector control strategies.

## Supplementary information

**Additional file 1: Text S1.** Test of the Permanet 3.0 using the Cup assay.

**Additional file 2: Figure S1.** Mortality 24-h post-exposure. The bars show the mean (± SE) proportion of mosquitoes that died following exposure to either the side of the Permanet 3.0 (red bars) or top of the Permanet 3.0 (blue bars).

**Additional file 3: Figure S2.** Blood-feeding behavior of mosquitoes. The bars show the mean (± SE) proportion of mosquitoes that successfully took a blood meal from a human host arm through either the side of the Permanet 3.0 (red bars) or the top of the Permanet 3.0 (blue bars).

**Additional file 4: Figure S3.** Mortality 24-h post-exposure based on blood-fed status. The bars show the mean (± SE) proportion of mosquitoes that died following either a successful or unsuccessful attempt to blood-feed on a human host arm while being exposed either the top of the Permanet 3.0 (left panel) or the side of the Permanet 3.0 (right panel).

## Data Availability

The datasets supporting the conclusions of this article are included within the article and its additional file.

## Abbreviations

LLIN: long-lasting insecticidal net
WHO: World Health Organization
CDC: Centers for Disease Control and Prevention
