# Supplementary material for: Use of alternative bioassays to explore the impact of pyrethroid resistance on LLIN efficacy
Source: Parasit Vectors. 2020 Apr 7;13:179. doi: 10.1186/s13071-020-04055-9 (PMC7140572; doi:10.1186/s13071-020-04055-9)
Supplement: Supplementary file 1 — Additional file 1: Text S1. Test of the Permanet 3.0 using the Cup assay. [file 13071_2020_4055_MOESM1_ESM.docx]

**Additional file 1: Text S1.**

The functional impact of insecticide resistance on the efficacy of LLINs

Marissa K. Grossman^1^, Shune V. Oliver^2,3^, Matthew B. Thomas^1^

^1^Department of Entomology, Pennsylvania State University, University Park, PA, USA

^2^Centre for Emerging Zoonotic and Parasitic Diseases, National Institute for Communicable Diseases of the National Health Laboratory Service, Johannesburg, South Africa

^3^Wits Research Institute for Malaria, MRC Collaborating Centre for Multi-disciplinary Research on Malaria, School of Pathology, Faculty of Health Sciences, University of the Witwatersrand, Johannesburg, South Africa.

Experiment S1

**Methods**

To test the functionality of the Permanet 3.0 in comparison to the Permanet 2.0, we followed the same experimental procedures as Experiment 2, the “cup” assay, but used a Permanet 3.0. We tested both the side of the Permanet 3.0, which contains 2.8g/kg deltamethrin (no PBO), and the top of the Permanet 3.0, which contains 4.0g/kg of deltamethrin plus 25 g/kg of PBO. For reference, the Permanet 2.0 contains only 1.8g/kg deltamethrin (no PBO).

**Results**

All mosquito strains tested (Senn-DDT, Fumoz-Base, and Fumoz-R) exhibited 100% mortality when exposed to the portion of the LLIN with PBO (Fig S1). All strains also exhibited decreased bloodfeeding on the PBO portion compared to the side of the Permanet 3.0 (Fig S2). Additionally, bloodfeeding did not rescue any strain from mortality when they contacted the PBO portion of the net (Fig S3).
