# Supplementary figures and images for: Use of alternative bioassays to explore the impact of pyrethroid resistance on LLIN efficacy
Source: Parasit Vectors. 2020 Apr 7;13:179. doi: 10.1186/s13071-020-04055-9 (PMC7140572; doi:10.1186/s13071-020-04055-9)

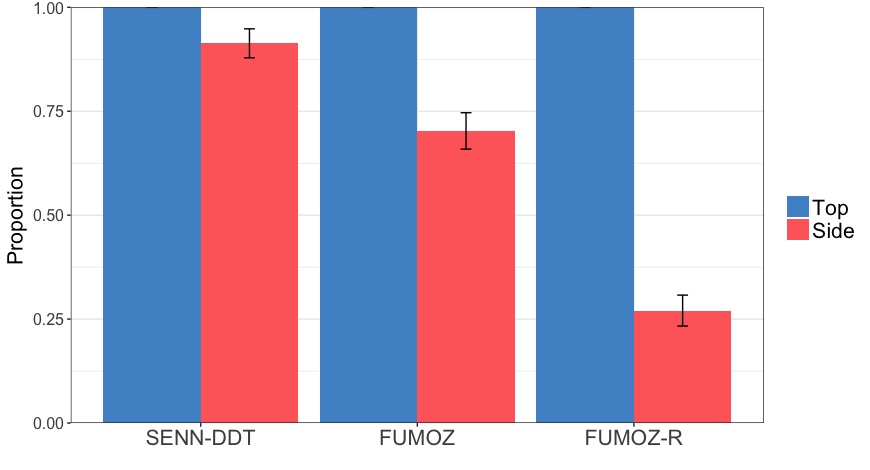

Supplement: Supplementary file 2 — Additional file 2: Figure S1. Mortality 24-h post-exposure. The bars show the mean (± SE) proportion of mosquitoes that died following exposure to either the side of the Permanet 3.0 (red bars) or top of the Permanet 3.0 (blue bars). [file 13071_2020_4055_MOESM2_ESM.jpeg]

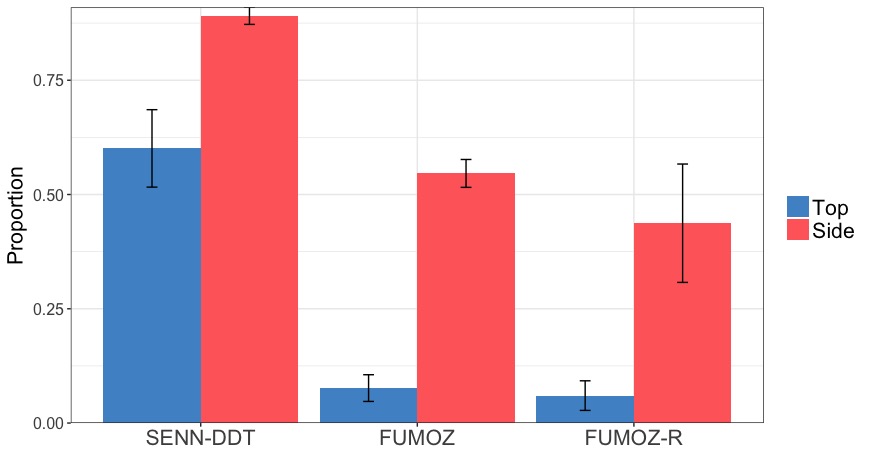

Supplement: Supplementary file 3 — Additional file 3: Figure S2. Blood-feeding behavior of mosquitoes. The bars show the mean (± SE) proportion of mosquitoes that successfully took a blood meal from a human host arm through either the side of the Permanet 3.0 (red bars) or the top of the Permanet 3.0 (blue bars). [file 13071_2020_4055_MOESM3_ESM.jpeg]

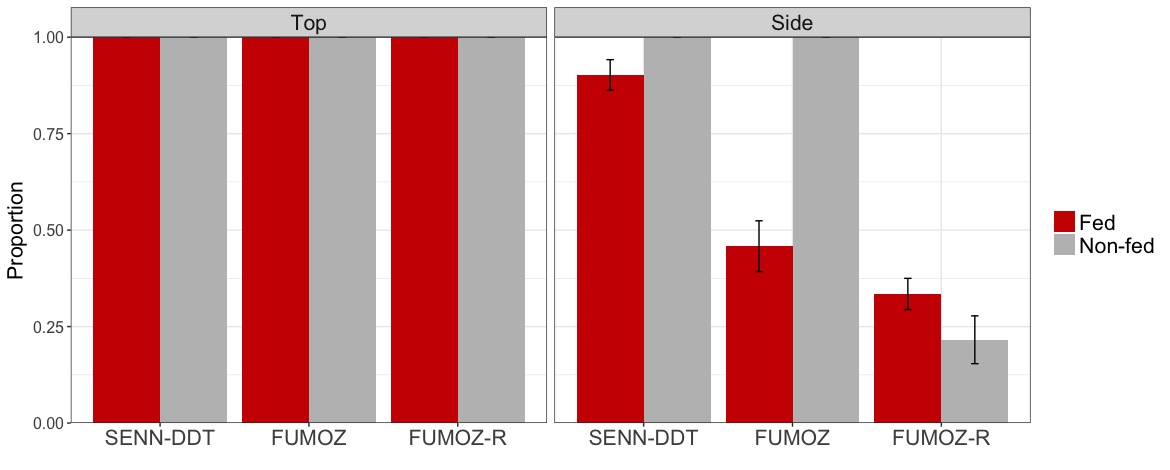

Supplement: Supplementary file 4 — Additional file 4: Figure S3. Mortality 24-h post-exposure based on blood-fed status. The bars show the mean (± SE) proportion of mosquitoes that died following either a successful or unsuccessful attempt to blood-feed on a human host arm while being exposed either the top of the Permanet 3.0 (left panel) or the side of the Permanet 3.0 (right panel). [file 13071_2020_4055_MOESM4_ESM.jpeg]
